# Increased incidence of postmyocardial infarction ventricular septal defects during Covid‐19 pandemic: A case series

**DOI:** 10.1111/jocs.16430

**Published:** 2022-03-21

**Authors:** M. D' Abramo, S. Saltarocchi, W. Vignaroli, E. Chourda, M. Vinciguerra, S. Romiti, G. Melina, E. Greco, F. Miraldi

**Affiliations:** ^1^ Department of Clinical, Internal Medicine, Anesthesiology and Cardiovascular Sciences Sapienza University of Rome Rome Italy; ^2^ Department of Clinical and Molecular Medicine, Cardiac Surgery Unit, Sant'Andrea Hospital Sapienza University of Rome Rome Italy

**Keywords:** complications, myocardial infarction, SARS‐Cov2, ventricular septal defect

## Abstract

**Introduction:**

Ventricular septal defect (VSD) is one of the mechanical complications of acute myocardial infarction (MI), whose incidence has been decreasing throughout the years because of the emergence of different reperfusion therapy strategies.

**Methods:**

We present a series of seven patients who underwent surgery for post‐MI VSD repair in our institution in the period between March 2020 and June 2021.

**Discussion:**

During the recent SARS‐COV2 pandemic, time to hospital admission increased due to patients being overcautious out of fear of exposing themselves to COVID‐19. The increased time to hospital admission, with associated late reperfusion therapy and delayed PCI, is closely related to an augmented incidence of post‐myocardial infarction mechanical complications such as ventricular septal defects. For this reason, we witnessed an increase in the incidence of post‐MI VSD.

**Conclusion:**

Fear of exposure to SARS‐COV2 in the medical environment was a major source of concern for all our patients. The target of hospital policy should be to reassure patients of freedom from COVID in the emergency department and cardiac wards in order to prevent such dreadful complications.

## 1 INTRODUCTION

Ventricular septal defect (VSD) is one of the possible mechanical complications of acute myocardial infarction (MI). VSD is generally located in the anterior or apical portion of the ventricular septum as a result of anterior MI (60%), or in the posterior portion as a result of posterior MI (40%).^1^ Its incidence dropped from 1% to 3% to 0.2% among MIs, as a result of the time reduction from infarction onset to proper care, as emphasized in the 2018 ESC/EACTS guidelines.^2^, ^3^ In fact, in patients with clinical suspicion of myocardial ischemia and ST‐elevation myocardial infarction (STEMI), reperfusion therapy needs to be initiated as soon as possible, to prevent complications.^4^

During the recent SARS‐COV2 pandemic, we witnessed a reduction in hospital admissions for MI, with studies demonstrating increased time‐to‐admission and a higher number of patients not suitable for revascularization at time of presentation.^5^ As foreseen by the literature,^6^, ^7^ as patients continued to avoid visiting hospitals out of fear of exposing themselves to COVID‐19, the incidence of mechanical complications of acute MI—including VSD—rose. We present seven cases of post‐MI VSD who presented to our hospital in the early period of the pandemic, between March 2020 and June 2021. Table 1 shows the main features of each patient.

**Table 1:** Patients' characteristics

Patient	Sex	Age	VSD location	EF (%)	Revascularization	IABP	Survival
#1	Male	78	Anterior	21	PCI on LAD	Yes	No
#2	Male	58	Posterior	45	PCI on RCA	Yes	Yes
#3	Male	75	Posterior	45	PCI on RCA	Yes	Yes
#4	Female	78	Posterior	50	PCI on RCA,	Yes	Yes
					CABG on LAD		
#5	Male	70	Posterior	50	ineffective PCI on LCX	Yes	No
#6	Male	71	Posterior	50	CABG on LAD, RCA, INT	Yes	
#7	Female	55	Anterior	40	PCI on LAD	Yes	Yes

Abbreviations: CABG, coronary artery bypass grafting; INT, intermediate artery; LAD, left anterior descending artery; PCI, percutaneous coronary intervention; RCA, right coronary artery.

### 1.1 Cases presentation

Patient #1: A 78‐year‐old gentleman reached our hospital, presenting with angina and dyspnea. TTE (transthoracic echocardiography) showed a reduced ventricular kinesis with an ejection fraction (EF) of 21% and an anterior VSD with a diameter of 20 mm. RV function was reduced with a Tricuspid annular plane systolic excursion (TAPSE) of 12 mm. Urgent coronary angiography revealed a completely occluded left anterior descending (LAD), which was treated with PCI (percutaneous coronary intervention). After the procedure, the patient suffered cardiac arrest and cardiopulmonary resuscitation (CPR) was initiated, completed with intra‐aortic balloon pump (IABP) support. Surgery was delayed for 12 days. The defect was closed with a single patch of bovine pericardium secured with U‐Stitches to the healthy endocardium. Unfortunately, the patient died 72 h later.

Patient #2: A 58‐year‐old man came to our attention with a diagnosis of posterior STEMI after worsening chest and epigastric pain. PCI was performed on a right coronary artery (RCA) lesion. Transesophageal echocardiography (TEE) demonstrated a reduced biventricular function, with an ejection fraction (EF) of 45%, TAPSE of 13 mm and the presence of a posterior VSD (*Q*_p_/*Q*_s_ = 2) with a large aneurysm of the posterior wall (40 mm in diameter). The patient initially refused surgery, but he returned to the emergency department 5 days later. Haemodynamic support was offered to the patient with IABP and surgery was performed 10 days later, correcting the VSD with a pericardial patch. The postoperative course was uneventful and he was discharged 20 days after the operation.

Patient #3: A 75‐year‐old male reached our hospital with a posterior STEMI. He underwent PCI, which treated an occlusion of the RCA. TEE showed a posterior VSD of 15 mm in diameter, a pseudoaneurysm of the ventricular septum (40 × 20 mm), and reduced EF (45%) and TAPSE (15 mm). An IABP was inserted to achieve haemodynamic stabilization before surgery. Surgical closure was performed 14 days later, and the defect was repaired with a pericardial patch. The patient was still dismissed 30 days after surgery.

Patient #4: A 78‐year‐old woman was admitted to our hospital with posterior STEMI. She underwent PCI to treat a proximal occlusion of the RCA due to a thrombus. Echocardiography showed a posterior VSD measuring 20 mm in diameter and a preserved ventricular function (EF 50%, TAPSE 19 mm). An IABP was inserted and the patient was stabilized. As in the previous case, surgery was postponed for 14 days, and the defect was closed with a pericardial patch. Concomitantly, the patient underwent LAD revascularization. The postoperative course was uneventful and the patient was discharged home two weeks after surgery.

Patient #5: A 70‐year‐old man was admitted to our hospital with a posterior STEMI. TEE showed a posterior VSD 12 mm in diameter, with a *Q*_p_/*Q*_s_ ratio of 2.2, and a preserved EF (50%), with mild reduction in right ventricular function (TAPSE 15 mm). The patient was stabilized with the support of an IABP, and PCI on a dominant circumflex artery (LCX) was performed—which was, however, ineffective. Delayed surgical repair was performed and the defect was closed with a pericardial patch. Postoperative TEE showed a residual left‐to‐right shunt with a *Q*_p_/*Q*_s_ of 1,7. Unfortunately, the patient died 20 days after the operation due to septic shock.

Patient #6: A 71‐year‐old male was admitted to our hospital and was diagnosed with posterior STEMI. Echocardiography showed a subtricuspid, 20 mm VSD, with a *Q*_p_/*Q*_s_ of 2.2 and an inferior ventricular aneurysm, with a preserved biventricular function (EF 50%, TAPSE 17 mm). The patient was stabilized with IABP. Coronary angiography showed critical lesions of the LAD, Intermediate Coronary Artery and RCA. Surgery was performed 14 days later; the defect was closed with a pericardial patch and concomitant CABG was performed. The postoperative course was uneventful and he was discharged 17 days after the operation. Intraoperative images for this case are shown in Figures 3, 4, 5.

Patient #7: A 55‐year‐old female came to our observation after an urgent PCI on LAD. After the procedure the patient had an episode of VF and cardiac arrest. Return of spontaneous circulation (ROSC) was obtained and IABP inserted. Echocardiogram showed a reduced ventricular function (EF 40%, TAPSE 14) and a VSD of 22 mm in diameter. Surgical closure with a circular pericardial patch with a continuous suture was subsequently performed. The postoperative course was complicated with pneumonia. She was dismissed 45 days after surgery.

## 2 DISCUSSION

**Figure 1:**
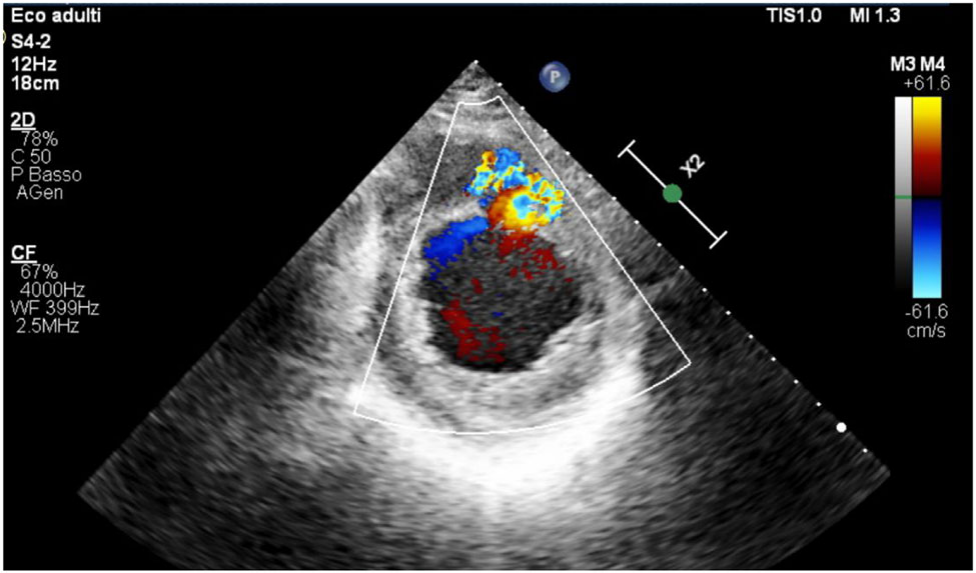
Transthoracic Doppler US showing blood flow across the ventricular septal defect

**Figure 2:**
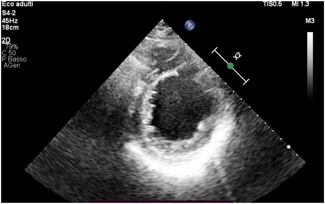
Ventricular septal defect seen with transthoracic echocardiography

The incidence of post‐MI VSDs decreased with the beginning of the era of early reperfusion therapy, dropping to 0.2% of all MIs.^3^ Generally, they are observed either in the first 24 h or 3–7 days post‐MI and are seen with transmural infarctions, mostly involving the LAD (60%). Their incidence is increased in the case of delayed coronary reperfusion.^8^, ^9^ Risk factors include advanced age, female sex, absence of smoking habit, hypertension, right ventricular infarction, and extensive MI.^10^ Two typical echocardiographic images of VSDs are shown in Figures 1 and  2.

**Figure 3:**
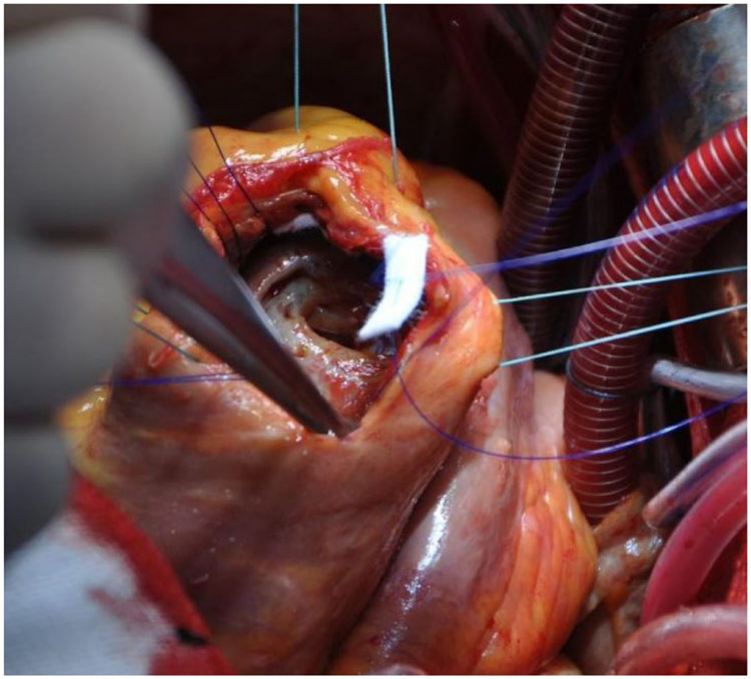
Intraoperative image showing ventricular septal defect accessed through a left ventriculotomy

**Figure 4:**
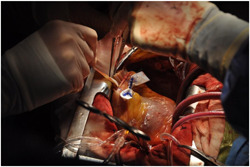
Intraoperative image of ventricular septal defect repair showing sizing of a bovine pericardium patch

**Figure 5:**
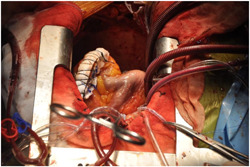
Final result of postmyocardial infarction ventricular septal defect repair showing closure of left ventriculotomy reinforced with polytetrafluoroethylene strips

Optimal timing for post‐MI VSD repair is still debated: in the case of early repair, there is an increased risk of recurrent ventricular rupture due to the absence of fibrosis at the margins of the defect and an associated mortality rate of 20%–40%, while in the case of delayed surgical repair the patient may experience the development of right ventricular failure and pulmonary hypertension, with hemodynamic instability and subsequent increased risk of death. IABP insertion may temporarily support haemodynamics, allowing for a delayed surgical repair.^1^ Figures 3, 4, 5 are intraoperative images that highlight the main phases of surgical repair of a post‐MI VSD.

Our management choice was based both on multidisciplinary team (MDT) discussion and on individual surgeon experience. This helped to decide on the best individualized approach in accordance with literature data by opting for procedures the surgeon was comfortable with.

During the global COVID‐19 pandemic, many patients did not reach the hospital at first signs of an adverse cardiac event, with an increased number of patients seeking medical advice with a certain delay.^11^, ^12^

In our series, we witnessed an unexpected prevalence of posterior VSD (71.4%). A possible explanation for this increased incidence may be the atypical presentation of inferior AMI, especially when the RV is involved. Most common symptoms are hypotension, nausea and vomiting, generally associated with epigastric pain, which often causes AMI to be misdiagnosed as “indigestion.”^13^ During the COVID pandemic, each of the patients included in our series deferred seeking medical care and only reached the hospital after days of thoracic pain and dyspnea, a symptomatology more related to VSD than AMI.

Fear of exposure to SARS‐COV2 was a major source of concern for all our patients, and it eventually led to delayed coronary reperfusion. We witnessed an increased incidence of VSD cases in our surgical practice during the pandemic, although outcomes were comparable to those of the pre‐COVID period. In the 5 years preceding the appearance of SARS‐COV2, with more than 2500 surgical procedures performed, we only had three cases (0.12%) of post‐MI VSD, while from March 2020 to June 2021 we treated seven VSD patients, amongst a total of 500 surgical procedures (1.4%). Demographic features did not change, with a prevalence of males in both groups (71% in the pre‐SARS‐COV2 era, 66% in the SARS‐COV2 period) and mean age of 66 y.o. in the first group (pre‐COVID) and of 70 y.o. in the second group. Clinical presentation did not change, except for timing.

Even when surgical repair is performed early, survival in cases of post‐MI VSD remains poor, with 30‐day mortality reaching 40%, more prevalent in posterior VSDs.^14^ In our case, mortality did not change significantly when compared to the pre‐SARS‐COV2 era (33% vs. 28%) and its incidence did not differ in cases of anterior or posterior VSD both in the pandemic era (1 vs. 1) and in the pre‐SARS‐COV2 period (1 death, anterior VSD). In the two cases when concomitant revascularization was performed, we have witnessed a better outcome in terms of mortality (0 vs. 2) and mean hospital stay (16  vs. 31 days).

## 3 CONCLUSION

Ventricular septal rupture is one of the most devastating mechanical complications of MI. Even if none of our patients tested SARS‐COV2 positive, and our surgical and cardiological units were COVID‐free, fear of exposure to the virus within the hospital environment kept them from reaching out for proper care. None of our patients consulted a general practitioner or a cardiologist at the onset of symptoms and only arrived at the hospital after days of ongoing dyspnea or thoracic pain. We believe this is one of the critical issues underlined by the recent pandemic, and considering the appearance of different COVID variants, every effort should be made to make patients feel safe with in‐person contact with healthcare providers.

## CONFLICTS OF INTEREST

The authors declare that there are no conflicts of interest.

## ETHICS STATEMENT

The authors declare that complete informed consent was obtained from patients for the publication of this study and accompanying images.
